# Photobiomodulation Mitigates PM_2.5_-Exacerbated Pathologies in a Mouse Model of Allergic Asthma

**DOI:** 10.3390/antiox13081003

**Published:** 2024-08-19

**Authors:** Jisu Park, Bo-Young Kim, Eun Jung Park, Yong-Il Shin, Ji Hyeon Ryu

**Affiliations:** 1 Research Institute for Convergence of Biomedical Science and Technology, Pusan National University Yangsan Hospital, Yangsan 50612, Gyeongnam, Republic of Korea; jiss5022@naver.com (J.P.); kimboyoung@pusan.ac.kr (B.-Y.K.); foreverpak1@nate.com (E.J.P.); 2 Department of Rehabilitation Medicine, School of Medicine, Pusan National University, Yangsan 50612, Gyeongnam, Republic of Korea

**Keywords:** asthma, ferroptosis, oxidative stress, particulate matter (PM_2.5_), photobiomodulation

## Abstract

Exposure to particulate matter (PM), especially PM_2.5_, is known to exacerbate asthma, posing a significant public health risk. This study investigated the asthma-reducing effects of photobiomodulation (PBM) in a mice model mimicking allergic airway inflammation exacerbated by PM_2.5_ exposure. The mice received sensitization with ovalbumin (OVA) and were subsequently treated with PM_2.5_ at a dose of 0.1 mg/kg every 3 days, for 9 times over 3 weeks during the challenge. PBM, using a 610 nm wavelength LED, was applied at 1.7 mW/cm^2^ to the respiratory tract via direct skin contact for 20 min daily for 19 days. Results showed that PBM significantly reduced airway hyperresponsiveness, plasma immunoglobulin E (IgE) and OVA-specific IgE, airway inflammation, T-helper type 2 cytokine, histamine and tryptase in bronchoalveolar lavage fluid (BALF), and goblet cell hyperplasia in PM_2.5_-exposed asthmatic mice. Moreover, PBM alleviated subepithelial fibrosis by reducing collagen deposition, airway smooth muscle mass, and expression of fibrosis-related genes. It mitigated reactive oxygen species generation, oxidative stress, endoplasmic reticulum stress, apoptotic cell death, ferroptosis, and modulated autophagic signals in the asthmatic mice exposed to PM_2.5_. These findings suggest that PBM could be a promising intervention for PM_2.5_-induced respiratory complications in patients with allergic asthma.

## 1. Introduction

Asthma is a chronic respiratory disease characterized by airway inflammation, variable airflow obstruction, airway hyperresponsiveness (AHR), and structural remodeling of the lungs. It affects millions of individuals worldwide, posing a significant health concern [1]. The pathogenesis of asthma is complex, involving a combination of genetic predispositions and environmental factors that contribute to the development and exacerbation of this respiratory disorder.

Exposure to particulate matter (PM), one of the prominent airborne pollutants, is associated with an elevated risk of incidence and exacerbations of the severity of pulmonary diseases like asthma. Its health effects are more aggravated in children being more vulnerable than adults [2]. Fine PM, with a diameter smaller than 2.5 µm (referred to as PM_2.5_), can penetrate the bronchioles and alveoli, trigger inflammation and oxidative stress, and impair respiratory functions [3]. Additionally, maternal exposure to elevated concentrations of PM_2.5_ during pregnancy increases the likelihood of asthma incidence in infants [4]. PM_2.5_ exposure can also cause chronic non-specific inflammatory respiratory diseases [5,6], leading to reversible airflow limitation, airway inflammation, airway remodeling, and increased AHR, thus complicating asthma management [7]. Therefore, understanding the relationship between PM exposure and asthma exacerbation, particularly emphasizing the importance of elucidating the underlying mechanisms involved and developing targeted therapeutic interventions, has gained increased research interest.

Photobiomodulation (PBM) is a form of light therapy that uses non-ionizing light sources, such as lasers, light emitting diodes (LEDs), or broadband light, in the visible and near-infrared spectrum. It is a non-invasive treatment approach that reduces pain and inflammation, promotes tissue repair, and improves overall cellular function [8,9]. The mechanism action of PBM involves the absorption of light energy by cellular components, such as mitochondria, which leads to increased ATP production, modulation of reactive oxygen species (ROS), and activation of intracellular signaling pathways [10]. These cellular responses result in anti-inflammatory, analgesic, and regenerative effects, making PBM a promising therapeutic approach for a wide range of medical conditions. This therapy is cost-effective, easy to use, and, most importantly, free from long-term side effects [11]. Therefore, PBM therapy has garnered wide recognition for its potential therapeutic benefits across various medical fields.

Drug therapy remains the primary approach for managing chronic respiratory diseases, such as asthma. Therefore, the key focus of the reported studies has been novel drugs to treat such conditions; nevertheless, the efficacy of PBM as an adjunctive therapy for respiratory disease treatment has also been recognized. Numerous studies have shown the effectiveness of PBM therapy in experimental models of respiratory conditions. Researchers have been actively investigating the potential therapeutic effects of PBM on asthma both in clinical settings and in experimental models. Using orange LED light at a wavelength of 610 nm, PBM therapy has been shown to exhibit anti-asthmatic effects by suppressing Th2 responses and bronchoconstriction-promoting substances through inhibition in the MAPK/NF-κB cascade [12]. Moreover, PBM therapy has been demonstrated to reduce inflammation by promoting an increase in regulatory T cell population [13] and regulating mast cell degranulation and IL-10 levels [14]. Furthermore, PBM has a beneficial effect on corticosteroid-resistant asthma mice [15]. Low-intensity laser PBM has proven to be both highly effective and safe in a study involving 220 patients with bronchial asthma [16]. In another study involving low-level laser acupuncture in 48 asthmatic children, 91.7% of patients experienced improved asthma control through a notable reduction in the breath condensate fractional exhaled nitric oxide level and a significant increase in spirometry parameters [17]. This concerted effort underscores a growing interest in exploring how PBM may offer novel treatment options for individuals with asthma, highlighting the need for further exploration and validation of its efficacy in respiratory conditions. Therefore, in the context of allergic asthma exacerbated by PM_2.5_ exposure, the use of PBM as a therapeutic intervention shows promise in modulating immune responses, reducing airway inflammation, and ameliorating oxidative stress-induced damage in the lungs.

The present study aimed to explore the effects of PBM on PM_2.5_ exposure-exacerbated allergic asthma in a mouse model. By assessing a range of parameters, including AHR, airway inflammation, T-helper type 2 (Th2) cytokines, bronchoconstrictor mediators, subepithelial fibrosis, endoplasmic reticulum (ER) stress induced by reactive oxygen species (ROS), apoptosis, ferroptosis and autophagic signals, the study sought to elucidate the potential anti-asthmatic mechanisms of PBM treatment. The study significantly advances the knowledge in this research field by demonstrating the effectiveness of PBM in alleviating and attenuating a broad range of adverse effects caused by PM_2.5_ exposure in allergic asthma. It not only highlights the potential of PBM as a promising therapeutic intervention for individuals with allergic asthma exposed to environmental pollutants like PM_2.5_ but also underscores the importance of considering multiple cellular and molecular pathways in the evaluation of treatment outcomes. Overall, the study provides valuable insights into the holistic effects of PBM on various pathological mechanisms associated with allergic asthma exacerbations induced by environmental factors like PM_2.5_.

## 2. Materials and Methods

### 2.1. PM_2.5_ Preparation

The PM_2.5_ (PM_2.5_-like; ERM-CZ110) used in the study was purchased from European Reference Materials (B-2400, European Commission, Geel, Belgium) and was obtained from the walls and sidewalks of a road tunnel in Poland. The specific manufacturing processes of PM_2.5_ samples are detailed in their corresponding certification reports [18]. This PM_2.5_ was certified for its water-soluble ions content, which includes Na^+^ (20.4 g/kg), K^+^ (3.3 g/kg), Ca^2+^ (44 g/kg), Mg^2+^ (1.8 g/kg), Cl^−^ (26.2 g/kg), NO_3_^−^ (7.8 g/kg), and SO_4_^2−^ (75 g/kg) [18]. The PM_2.5_ was diluted in phosphate-buffered saline (PBS) and sonicated before use.

### 2.2. Mouse Model of Asthma and PM_2.5_ Exposure

**Figure 1:**
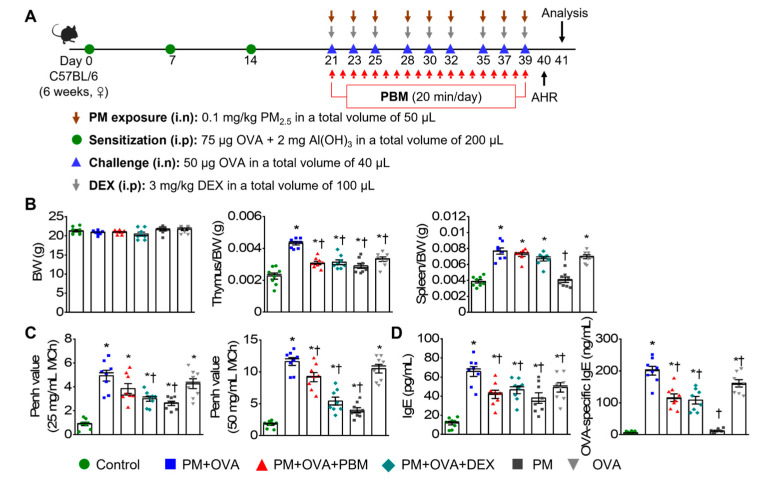
Inhibitory effects of PBM on the induction of airway hyperresponsiveness (AHR) and plasma IgE in a PM_2.5_-exposed asthma exacerbation model. (**A**) Establishment of an allergic asthma exacerbation mouse model induced by PM_2.5_ exposure. A timeline describing the asthma exacerbation model induction and PBM treatment. (**B**) Measurement of body weight, thymus-to-body-weight ratio, and spleen-to-body-weight ratio on the final day of the experiment. (**C**) Assessment of AHR to methacholine (MCh) at concentrations of 25 and 50 mg/mL. (**D**) Measurement of total immunoglobulin E (IgE) and ovalbumin (OVA)-specific IgE in plasma. Data are shown as the mean ± SEM (*n* = 8). * p < 0.05 compared with control. ^†^ *p* < 0.05 compared with PM + OVA. i.n., intranasal injection; i.p., intraperitoneal injection; BW, body weight; PM, particulate matter; OVA, ovalbumin; PBM, photobiomodulation; DEX, dexamethasone.

All animal studies were sanctioned by the Institutional Animal Care and Use Committee of Pusan National University Yangsan Hospital and conducted in compliance with the National Institute of Health Guidelines (IACUC No.: 2021-030-A1C0(0)). The mice were assigned to six groups with 7−8 mice in each group, labeled as control, PM + OVA, PM + OVA + PBM, PM + OVA + DEX, PM, and OVA. The asthma model was established following a method outlined in a prior investigation [19]. Briefly, on days 0, 7, and 14, each mouse was sensitized by intraperitoneal injection of 75 µg OVA (BioVender, Asheville, NC, USA) in 200 µL PBS containing 2 mg aluminum hydroxide (Imject Alum; Thermo Scientific, Rockford, IL, USA). The mice were subjected to an intranasal challenge of 50 µg OVA in PBS three times a week for 3 weeks following isoflurane anesthesia induction (Hana Pharm Co., Ltd., Hwaseong, Republic of Korea; 2% induction and 1.5% maintenance, in 80% N_2_O and 20% O_2_). Control mice were sensitized and challenged with PBS at the corresponding times. Dexamethasone (3 mg/kg every 3 days; DEX; Sigma-Aldrich, St. Louis, MO, USA) was intraperitoneally administered 2 h before each OVA challenge. For PM-induced aggravation of asthma, mice were anesthetized with isoflurane and intranasally instilled with PM_2.5_ (0.1 mg/kg/day) in 50 µL of PBS three times a week for 3 weeks before challenge (Figure 1A). The concentration of PM_2.5_ used in the study was determined based on previous studies [20,21]. On the day of the procedure, measurements were taken for the spleen and thymus weights.

### 2.3. Photobiomodulation Using Light-Emitting Diode (LED)

An LED device (dimensions: 4.9 × 4.9 × 1.3 cm, Color Seven Co., Seoul, Republic of Korea) was used for PBM with the following specifications: peak wavelength, 610 nm (full width at half maximum, 24 nm); power intensity, 1.7 mW/cm^2^; energy density, 2.0 J/cm^2^; electrode surface area, 1.6 cm; electrode spot size, 4 mm in diameter. The PBM protocol was based on a previous study [12]. Briefly, light stimulation was administered by placing probes onto the respiratory tract via direct skin contact once daily for 20 min after each OVA or PBS challenge under isoflurane anesthesia. The control group underwent the same anesthesia duration without PBM (Figure 1A).

### 2.4. Measurement of AHR

AHR to inhaled methacholine (MCh; Sigma) was assessed 24 h after the last challenge using whole-body plethysmography (OCP 3000, Allmedicus, Gyeonggi, Republic of Korea), following the established protocol [19]. The mice were subjected to 25 and 50 mg/mL concentrations of MCh for 10 min through a nebulizer (HARVARD73-1963; Harvard Apparatus, Holliston, MA, USA), and they were promptly placed back into their enclosures, with measurements being taken 150 s later. Enhanced pause (Penh), an indicator of airway resistance, was calculated using the average pressure recorded in the plethysmography chamber.

### 2.5. Bronchoalveolar Lavage Fluid (BALF) and Inflammatory Cells

The collection of BALF and subsequent differential cell counts were conducted following established procedures [19]. Mice were euthanized with a lethal dose of avertin tribromoethanol (Sigma-Aldrich Chemical Co., St. Louis, MO, USA).

A tracheostomy tube was used to flush the lung specimen with 1 mL of sterile cold PBS. BALF samples were centrifuged at 300× *g* for 10 min at 4 °C, and total bronchoalveolar lavage cells were enumerated using a hemocytometer. Cytospin (Thermo Shandon, Pittsburgh, PA, USA) was employed to attach differential cells to slides, which were subsequently stained with Diff-Quick (Sysmex International Reagents, Kobe, Japan). A total of 300 cells were counted under microscopy to determine the differential cell counts. BALF supernatants were stored at −80 °C until cytokine analysis.

### 2.6. Enzyme-Linked Immunoassay (ELISA)

Determination of total and OVA-specific IgE concentrations in plasma was assessed by sandwich ELISA (Mouse IgE ELISA kit, Bethyl Laboratories, Montgomery, TX, USA; anti-ovalbumin IgE ELISA kit, Cayman Chemical, Ann Arbor, MI, USA), as described previously [22]. Determination of IL-4, IL-5, IL-13, and histamine concentration in BALF was determined by sandwich ELISA (Th2 cytokines, R&D System, Minneapolis, MN, USA; histamine, Enzo Life Sciences, Ann Arbor, MI, USA) following the guidelines provided by the manufacturer [22]. Additionally, the levels of mast cell tryptase in BALF were also analyzed using an ELISA kit (Cusabio Biotech, Wuhan, China), as described in a previous study [23]. The optical density at 450 nm was measured with a microplate reader (SpectraMax iD5; Molecular Devices, San Jose, CA, USA).

### 2.7. Lung Histology and Immunohistochemistry

The lung tissues were fixed, embedded in paraffin, and 5 µm sections were prepared from the blocks. Lung inflammation was assessed via hematoxylin and eosin (H&E) staining, mucus production was analyzed using the periodic acid–Schiff (PAS) staining kit (Millipore, Billerica, MA, USA), and collagen accumulation was evaluated by Masson’s trichrome (MT) staining kit (Polysciences, Warrington, PA, USA) and Picro Sirius Red staining (Abcam, Cambridge, UK). Lung inflammation severity, mucin-positive goblet cells, and collagen deposition were scored using established grading systems [24,25,26]. Imaging was performed under a digital microscope (Axio Scan.Z1; Carl Zeiss, Jena, Germany), tissue analysis was conducted by three separate evaluators, and the outcomes were averaged. To determine airway smooth muscle volume, lung tissue samples were deparaffinized and kept overnight at 4 °C with monoclonal anti-actin and anti-smooth muscle actin (α-SMA)-FITC antibody (1:500; Sigma). The α-SMA immunostaining area was visualized using a confocal laser scanning microscope (LSM 900; Carl Zeiss, Germany) and quantified to assess airway smooth muscle layer thickness [27]. Immunohistochemical detection of 8-hydroxy-2′-deoxyguanosine (8-OHdG) in tissue slides was performed by antigen retrieval, and endogenous peroxidase was inactivated by treatment with 3% hydrogen peroxide. The slides were washed thrice in PBS and incubated with blocking buffer (10% normal goat serum in PBS) for 60 min at room temperature. The slides were incubated with 8-OHdG (Abcam) antibody overnight at 4 °C. Subsequently, the slides were incubated with horseradish peroxidase-conjugated goat anti-rabbit IgG antibody (DAKO, Carpinteria, CA, USA) for 60 min to detect immunoactivity, followed by detection using a DAB solution kit (DAKO). Hematoxylin was used as a counterstain. The stained specimens were examined using a digital microscope (Axio Scan.Z1; Carl Zeiss). Five randomly selected sections were quantified using ImageJ software (version 1.8.0; National Institute of Health, Bethesda, MD, USA).

### 2.8. Terminal Deoxynucleotidyl Transferase dUTP Nick-End Labeling (TUNEL)

TUNEL staining was performed to evaluate the degree of apoptosis using the DeadEndTM Fluorometric TUNEL System kit (Promega, Madison, WI, USA) following the manufacturer’s instructions and the procedure described in a previous study [28].

The lung sections were treated with 4′,6-diamidino-2-phenylindole (DAPI, 5 μg/mL), and cells positive for TUNEL staining were observed under a fluorescent microscope (K1-Fluo; Nanoscope Systems, Daejeon, Republic of Korea).

### 2.9. Perl’s Prussian Blue (PPB) Staining for Iron Accumulation in Lung Tissues

Iron deposition in lung tissue was detected using PPB staining, following previously established methods [29]. Briefly, lung tissue embedded in paraffin was subjected to deparaffinization followed by hydration with distilled water. A staining solution was prepared by mixing potassium ferrocyanide (10%; Sigma-Aldrich) and hydrochloric acid (20%; Bio Basic Inc., Toronto, ON, Canada) in equal amounts. The lung tissue slides were immersed in the prepared solution for 30 min on a shaker, followed by three washes with distilled water. Subsequently, the slides were counterstained with a nuclear fast red solution (Sigma-Aldrich) for 5 min and rinsed twice with distilled water. Subsequently, the slides were affixed using a resin-based mounting material following dehydration. PPB-stained samples were evaluated using a semiquantitative scoring system as per the following criteria: 0 = absence of staining, 1 = minimal staining, 2 = mild staining, 3 = moderate staining, and 4 = intense staining [30].

### 2.10. Measurement of Total Free Radical Activity in Lung Tissue

Lung tissue samples were used to measure the total levels of ROS/reactive nitrogen species (RNS) by employing the OxiSelect™ ROS/RNS assay kit from Cell Biolabs (San Diego, CA, USA), following the provided instructions. Briefly, the lung samples were homogenized using radioimmunoprecipitation assay (RIPA) buffer (Thermo Fisher Scientific, Rockford, IL, USA) to obtain tissue lysates, which were then centrifuged at 12,000*× g* for 15 min at 4 °C after being sonicated on ice for 1 min. Fluorescence was evaluated under a microplate reader (SpectraMax iD5).

### 2.11. Measurement of Malondialdehyde (MDA) Levels in Lung Tissue

MDA concentrations in lung samples were determined using OxiSelect™ TBARS assay kit (Cell Biolabs, San Diego, CA, USA) following a previously described method [23]. Lung tissue samples were homogenized to a concentration of 50 mg/mL in PBS and then supplemented with Butylated Hydroxytoluene (BHT; 1×) to prevent further oxidation during the process.

After centrifuging the lung tissues at 10,000× *g* for 10 min at 4 °C, supernatant containing the soluble components was carefully collected for the assay. MDA standards ranging from 0 to 125 μM were prepared by serially diluting in distilled water. Subsequently, 100 μL of samples or MDA standards were transferred to microcentrifuge tubes, to which 100 μL of sodium dodecyl sulfate (SDS) lysis solution was added, thoroughly mixed, and then incubated at 20–25 °C for 5 min. Next, 250 μL of thiobarbituric acid (TBA) was added, followed by thorough mixing and incubation at 95 °C for 45 min. After cooling to 20–25 °C for 5 min, the samples were centrifuged at 800× *g* at 20–25 °C for 15 min, and the resulting supernatants were transferred into clean tubes. Subsequently, standards and samples, each at a volume of 200 μL, were added to individual wells of a 96-well plate. The optical density at 532 nm was measured with a microplate reader (SpectraMax iD5).

### 2.12. Measurement of Glutathione (GSH) Content in Lung Tissue

The glutathione concentration in lung tissue was measured using the OxiSelect™ Total glutathione (GSSG/GSH) assay kit (Cell Biolabs) following the manufacturer’s instructions. Briefly, lung tissue lysates were prepared by treating the samples with metaphosphoric acid (MPA) for deproteination. Then 100 μL of lung samples or GSH standards were transferred to a 96-well plate, and 25 μL 1× GSH reductase solution and 25 μL 1× NADPH solution were added. Subsequently, 50 μL of 1× chromogen was added to the reaction mixture, and the absorbance was measured at 405 nm using a microplate reader (SpectraMax iD5).

### 2.13. Quantification of Total Calcium Content in Lung Tissue

The calcium content in lung tissue was measured using the Calcium Detection Assay kit (Abcam) following a previously described method [31]. Initially, 50 mg lung tissues were homogenized in 200 μL calcium assay buffer and sonicated for 10 s in an ice bath. The homogenate was then centrifuged at 12,000× *g* for 5 min, and the supernatant was transferred to a clean tube and used as the tissue lysate. Calcium standards were prepared in serial dilutions ranging from 0 to 1 mM in distilled water. To initiate the reaction, 50 μL calcium standard or samples were mixed with 90 μL chromogenic reagent and 60 μL calcium assay buffer in a 96-well plate. The plate was then incubated for 10 min at 20–25 °C in the dark. The absorbance was read at 575 nm using a microplate reader (SpectraMax iD5).

### 2.14. RNA Isolation and Real-Time Quantitative Polymerase Chain Reaction (RT-qPCR)

RT-qPCR was performed using the protocol detailed in a prior investigation [9]. Total RNA was extracted from the left lung of mice using TRIzol reagent (Invitrogen, Waltham, MA, USA). Subsequently, 2 μg total RNA was utilized for the synthesis of the first cDNA using the amfiRivert Platinum cDNA synthesis master mix (GenDEPOT, Barker, TX, USA) according to the manufacturer’s instructions. Quantitative polymerase chain reaction was performed using FastStart Essential DNA Green Master (Roche Diagnostics, Mannheim, Germany). The quantification of target genes expression was determined using the 2−ΔΔCt comparative method with normalization against glyceraldehyde 3-phosphate dehydrogenase (*Gapdh*). Primer sequences are listed in Supplementary Table S1.

### 2.15. Western Blot Analyses

Western blotting was performed following established methods [19]. Briefly, lung tissues were homogenized in ice-cold RIPA buffer containing a protease inhibitor cocktail (GenDEPOT). The homogenates were sonicated and then incubated for 30 min on ice. After centrifugation (15,000× *g* for 15 min) the supernatant containing the protein samples were moved to a microcentrifuge tube, and the protein concentration was assessed using a PierceTM BCA Protein Assay Kit (Thermo Fisher Scientific).

The lung lysates were subjected to SDS-polyacrylamide gel electrophoresis and transferred onto polyvinylidene difluoride membranes (Millipore, Darmstadt, Germany), and blocked with 5% nonfat dry milk overnight at 4 °C with the specified primary antibodies: anti-superoxide dismutase 1 (SOD1; Abcam), anti-peroxiredoxin 4 (Prdx4; Abcam), anti-protein kinase R-like ER kinase (PERK; Bioworld Technology, Minneapolis, MN, USA), anti-p-PERK (Bioworld Technology), anti-eukaryotic initiation factor 2α (eIf2-α; Bethyl Lab, Montgomery, TX, USA), p-eIF2-α (Bioworld Technology), anti-activating transcription factor (ATF4; Bioworld Technology), anti-C/EBP homologous protein (CHOP; Bioworld Technology), anti-poly ADP-ribose polymerase (PARP; Cell Signaling, Danvers, MA, USA), anti-Bcl2-associated X (Bax; Cell Signaling), anti-B-cell lymphoma protein 2 (Bcl2; Cell Signaling), anti-cleaved caspase-3 (Cell Signaling), anti-4-hydroxynoneal (4-HNE; R&D system), anti-glutathione peroxidase 4 (GPX4; Bioworld Technology), anti-solute carrier family 7 member 11 (SLC7A11; LSBio, Seattle, WA, USA), anti-heme oxygenase-1 (HO-1; Enzo life sciences, Farmingdale, NY, USA), anti-microtubule-associated protein 1A/1B-light chain 3B (LC3B; Cell Signaling), anti-autophagy related 3 (ATG3; Cell Signaling), ATG5 (Cell Signaling), ATG7 (Cell Signaling), anti-beclin-1 (Cell Signaling), and β-actin (Sigma-Aldrich). The membranes were then washed three times with Tris-buffered saline containing Tween-20 and incubated with the appropriate horseradish peroxidase-conjugated secondary antibodies (ENZO Life Sciences, Farmingdale, NY, USA) at 20–25 °C for 1 h. The HRP reaction was performed using an enhanced chemiluminescence kit (Amersham Pharmacia, Piscataway, NJ, USA), and the resulting chemiluminescence signal was captured with Amersham ImageQuant 800 (Cytiva, Marlborough, MA, USA). Each band was quantitatively determined using the ImageJ software (U. S. National Institutes of Health, Bethesda, MD, USA). The levels of relative proteins were confirmed with β-Actin acting as the loading control.

### 2.16. Statistics

Data are presented as the mean ± standard error of the mean (SE). Differences among groups were analyzed using either the one-way ANOVA/Bonferroni test or the Kruskal–Wallis/Mann–Whitney test, selected based on the results of the normality test (OriginPro 2020b software, OriginLab Corp., Northampton, MA, USA). A *p*-value of <0.05 was considered statistically significant.

## 3. Results

### 3.1. PBM Alleviated the Exacerbation of AHR and IgE Production Caused by PM_2.5_ Exposure in Allergic Asthma

In an asthma exacerbation mouse model induced by exposure to PM_2.5_, the anti-asthmatic effects of PBM were evaluated. The experimental groups had no significant difference in total body weight (BW) on day 41. In contrast, the thymus weight increased significantly in the PM + OVA group compared to that in the control group, which was notably reduced by treatment with PBM and DEX (Figure 1B). The spleen weight increased in all experimental groups except the PM group compared to the control group. However, the PBM did not reduce the increased weight of the spleen (Figure 1B). After the MCh challenge, the PM + OVA group demonstrated a progressive increase in Penh enhancement, which was dose-dependent. Treatment with PBM and DEX notably reduced the elevated Penh values observed in the PM + OVA group (at an MCh concentration of 50 mg/mL). Furthermore, Penh values in the asthma exacerbation group induced by PM did not show a significant difference compared to those in the OVA-alone treatment group (Figure 1C). In the PM + OVA group, plasma concentrations of total IgE and OVA-specific IgE were notably elevated. However, treatment with DEX and PBM significantly reduced these levels, with no significant difference observed between DEX and PBM treatments (Figure 1D). The IgE concentrations in the OVA group were notably lower compared to those in the OVA + PM group.

### 3.2. PBM Reduced the Exacerbation of Airway Inflammation and Th2 Responses Caused by PM_2.5_ Exposure in Allergic Asthma

**Figure 2:**
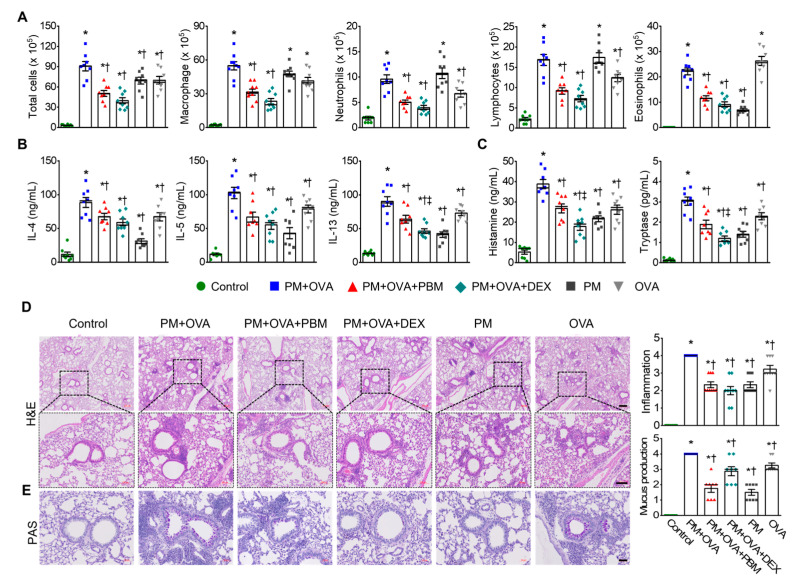
Inhibitory effects of PBM on the elevation of allergic airway inflammation and goblet cell metaplasia in a PM_2.5_-exposed asthma exacerbation model. (**A**) Measurement of total and differential inflammatory cell counts (macrophage, neutrophils, lymphocytes, and eosinophils) in bronchoalveolar lavage fluid (BALF). (**B**) Evaluation of Th2 cytokines including interleukin (IL)-4, IL-5, and IL-13 in BALF. (**C**) Assessment of histamine and mast cell tryptase in BALF. (**D**) Representative images of H&E staining revealed the infiltration of inflammatory cells in lung tissues. Scale bar represents 200 µm (up) and 100 µm (down). The bar graphs represent the summarized score of inflammation. (**E**) Goblet cells secreting mucus in lung tissues were identified using PAS staining. The bar graphs represent the number of PAS-reactive airway epithelial cells. Scale bar represents 50 µm. The bar graphs represent the summarized scores of PAS-positive mucus-producing cells. Data are shown as the mean ± SEM (*n* = 8). * p < 0.05 compared with control. ^†^ *p* < 0.05 compared with PM + OVA. ^‡^ *p* < 0.05 compared with PM + OVA + PBM.

Next, we assessed the potential effect of PBM treatment on allergic airway inflammation in asthma exacerbation induced by PM_2.5_ in BALF and lung tissue samples. BALF analysis revealed a significant increase in total cell count, macrophages, neutrophils, lymphocytes, and eosinophils in the PM + OVA group. Treatment with DEX and PBM attenuated these elevations with no significant differences between the two treatment groups (Figure 2A). Th2 cytokines in BALF, including IL-4, IL-5, and IL-13 were markedly increased in the OVA + PM group; however, they were significantly reduced in the DEX and PBM groups (Figure 2B). The concentrations of histamine and tryptase were significantly elevated in the PM + OVA group (Figure 2C). In contrast, these increases were significantly reduced in the PBM and DEX treatment groups, with a more pronounced effect seen in the DEX group than in the PBM group. Histological analyses of lung tissues showed increased inflammatory cell infiltration around the bronchioles in the PM + OVA group compared to the control group. The PM_2.5_-induced increased lung tissue inflammation in asthmatic mice was markedly reduced in those treated with PBM and DEX (Figure 2D). Goblet cell hyperplasia was elevated in the PM + OVA mice compared to the control mice. Treatment with PBM and DEX markedly decreased the in the proportion PAS-reactive airway epithelial cells triggered by exposure to PM_2.5_ (Figure 2E). Overall, PBM demonstrated comparable efficacy to DEX in reducing inflammation and mucus production in the lung tissues of mice exposed to PM_2.5_ during asthma exacerbation.

### 3.3. PBM Attenuated the Exacerbation of Subepithelial Fibrosis Caused by PM_2.5_ Exposure in Allergic Asthma

**Figure 3:**
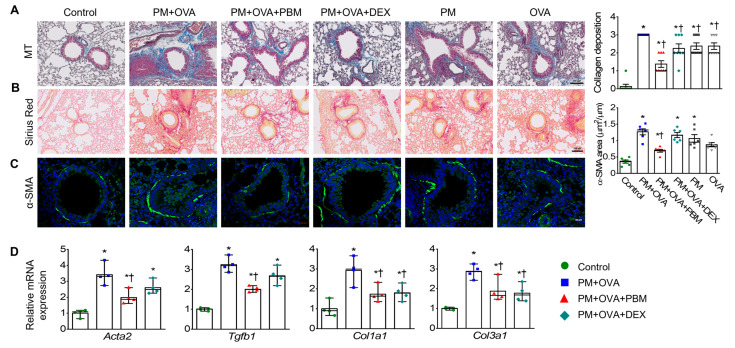
Inhibitory effects of PBM on the elevation of subepithelial fibrosis in a PM_2.5_-exposed asthma exacerbation model. Representative histological images showing (**A**) lung collagen fiber (Masson’s trichrome staining) and (**B**) collagen deposition (Sirius Red staining) in the lung tissues. Scale bar represents 100 µm. The bar graphs represent the summarized scores of collagen fiber deposition (*n* = 8). (**C**) Representative images of α-smooth muscle actin (α-SMA) and FITC expression, as determined by immunohistochemistry, in bronchioles of similar size. Scale bar represents 20 µm. The bar graphs represent the area of α-SMA staining per micrometer length of the bronchiolar basement membrane (µm^2^/µm; *n* = 6). (**D**) Detection of the mRNA levels of *Acta2*, *Tgfb1*, *Col1a1*, and *Col3a1* in lung tissues using qRT-PCR (*n* = 4). Data are shown as the mean ± SEM. * p < 0.05 compared with control. ^†^ *p* < 0.05 compared with PM + OVA.

Subepithelial fibrosis is linked to the severity of asthma, with higher levels of collagen exhibited in the airway walls of individuals with moderate to severe asthma compared to those with a milder form of the condition [32]. Consequently, we assessed different parameters related to subepithelial fibrosis in lung tissues to examine the effect of PM exposure on subepithelial fibrosis during asthma exacerbation. Analysis of collagen accumulation using MT and Sirius red staining revealed a significant increase in collagen fiber deposition in the PM + OVA group. Remarkably, both the PM + OVA + PBM and PM + OVA + DEX groups exhibited substantial reductions in collagen deposition, showing effects similar to those of DEX or PBM treatment (Figure 3A,B). Assessment of airway smooth muscle mass using alpha-smooth muscle actin (α-SMA) immunostaining showed a significant reduction in the increased airway smooth muscle mass in PM + OVA mice treated with PBM (Figure 3C). In contrast, DEX did not affect the increased airway smooth muscle thickness.

Subsequently, the expression of fibrosis-associated genes was measured in lung tissues. The PM + OVA group exhibited elevated mRNA levels of *Acta2* and *Tgfb1*, which were significantly reduced by PBM treatment but not by DEX. Additionally, the increased mRNA levels of *Col1a1* and *Col3a1* in the PM + OVA group were significantly decreased in both PM + OVA + PBM and PM + OVA + DEX groups (Figure 3D).

### 3.4. PBM Mitigated the Exacerbation of ROS-Mediated ER Stress Caused by PM_2.5_ Exposure in Allergic Asthma

**Figure 4:**
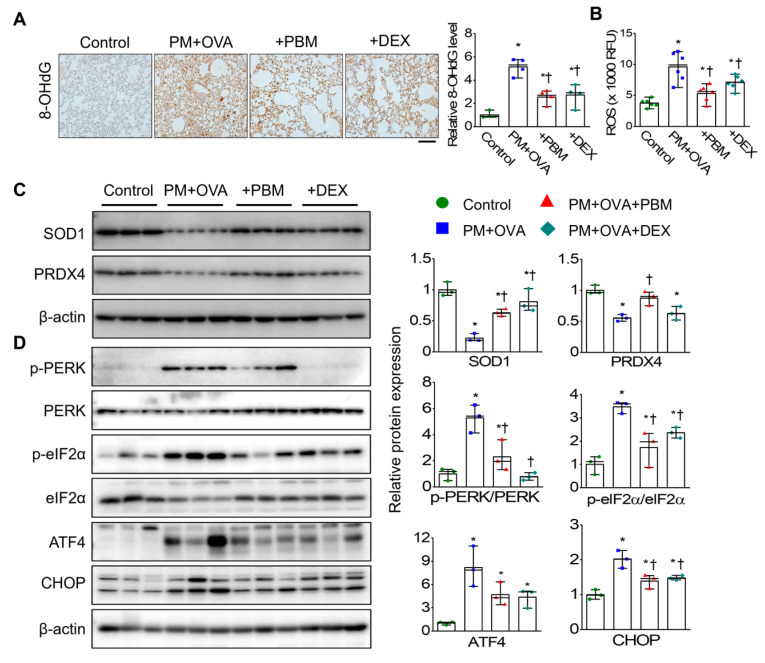
Inhibitory effects of PBM on the ROS-mediated ER stress in a PM_2.5_-exposed asthma exacerbation model. (**A**) Representative lung sections were stained with antibody specific for 8-hydroxy-2′-deoxyguanosine (8-OHdG). Bar graphs represent the quantification of positive areas of 8-OHdG in each experimental group (*n* = 4). Scale bar represents 100 µm. (**B**) ROS levels in lung tissue were measured in relative fluorescence units (RFU). (**C**) Protein expression of superoxide dismutase 1 (SOD1) and peroxiredoxin 4 (PRDX4). (**D**) ER stress markers (PERK, eIF2α, ATF4, and CHOP) in lung tissues by Western blotting. β-actin was used as a loading control. Bar graphs represent the quantification protein expression (*n* = 3). Data are shown as the mean ± SEM. * p < 0.05 compared with control. ^†^ *p* < 0.05 compared with PM + OVA.

To investigate the effect of PBM on oxidative stress induced by PM in allergic asthma, oxidative DNA damage was evaluated in lung tissue. The expression of 8-OHdG was significantly higher in the bronchiolar and alveolar epithelia of the PM + OVA group than in the control group. Moreover, both the PM + OVA + PBM and PM + OVA + DEX groups showed significant reductions in 8-OHdG expression, demonstrating similar effects with DEX or PBM treatment (Figure 4A). ROS plays a crucial role in allergic asthma by triggering airway inflammation and oxidative stress [33,34], which can worsen symptoms and exacerbate the condition. ROS activates inflammatory pathways, leading to increased mucus production, airway constriction, and airway remodeling in allergic asthma [34]. Thus, to evaluate the effects of PBM on ROS levels during asthma exacerbation induced by PM_2.5_ exposure, we measured ROS levels in the lung tissues of each experimental group. ROS levels in lung tissue were higher in the PM + OVA group than those in the control (Figure 4B). The PM + OVA + PBM group showed approximately a 45% decrease in ROS levels, while the PM + OVA + DEX group exhibited a 26% reduction compared to that in the PM + OVA group. 

The expression levels of oxidative stress markers, such as SOD1 and PRDX4, were significantly decreased in the PM + OVA group compared to those in the control group. However, the decreased expression of these oxidative stress markers was notably increased in the PM + OVA + PBM group (Figure 4C). Furthermore, PBM as well as DEX alleviated the increased expression of ER stress-associated proteins (PERK, eIF2α, ATF4, and CHOP) (Figure 4D).

### 3.5. PBM Inhibited the Exacerbation of Apoptosis, Ferroptosis, and Autophagic Signals Caused PM_2.5_ Exposure in Allergic Asthma

**Figure 5:**
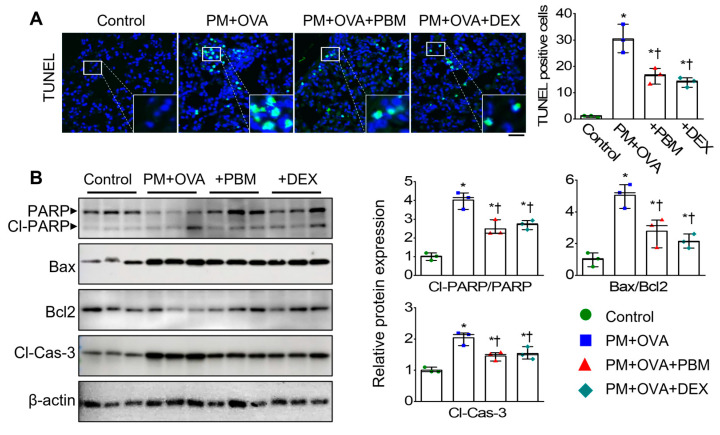
Inhibitory effects of PBM on the cell death in a PM_2.5_-exposed asthma exacerbation model. (**A**) Representative immunofluorescence for TUNEL (green) and DAPI (blue) staining. Scale bar represents 50 µm. Bar graphs represent TUNEL (+)/DAPI (+) cells in the lung tissues (*n* = 3). (**B**) Apoptotic markers in lung tissues. Bar graphs represent the quantification protein expression (*n* = 3). Bar graphs represent the quantification protein expression (*n* = 3). Data are shown as the mean ± SEM. * p < 0.05 compared with control. ^†^ *p* < 0.05 compared with PM + OVA.

Excessive inflammation and oxidative stress can trigger cell death [35]. To investigate the effect of PBM on cell death signaling in asthma exacerbation induced by PM_2.5_ exposure, we assessed the expression levels of apoptosis, ferroptosis, and autophagic signals. TUNEL staining on lung sections performed to assess the influence of PBM on pulmonary cell death by PM and OVA challenge revealed a significant increase in apoptotic cell numbers in the PM + OVA group compared to those in the control group (Figure 5A). However, the increased apoptotic cell numbers were significantly reduced in the PBM-treated group. Moreover, analysis of the expression of apoptotic markers in lung tissues revealed that cleavage PARP (Cl-PARP), BAX/BCL-2 ratio, and cleavage of caspase 3 (Cl-Cas 3) were upregulated in the PM + OVA mice (Figure 5B). In contrast, the expression of these apoptotic proteins was significantly decreased in the PBM and DEX-treated mice.

**Figure 6:**
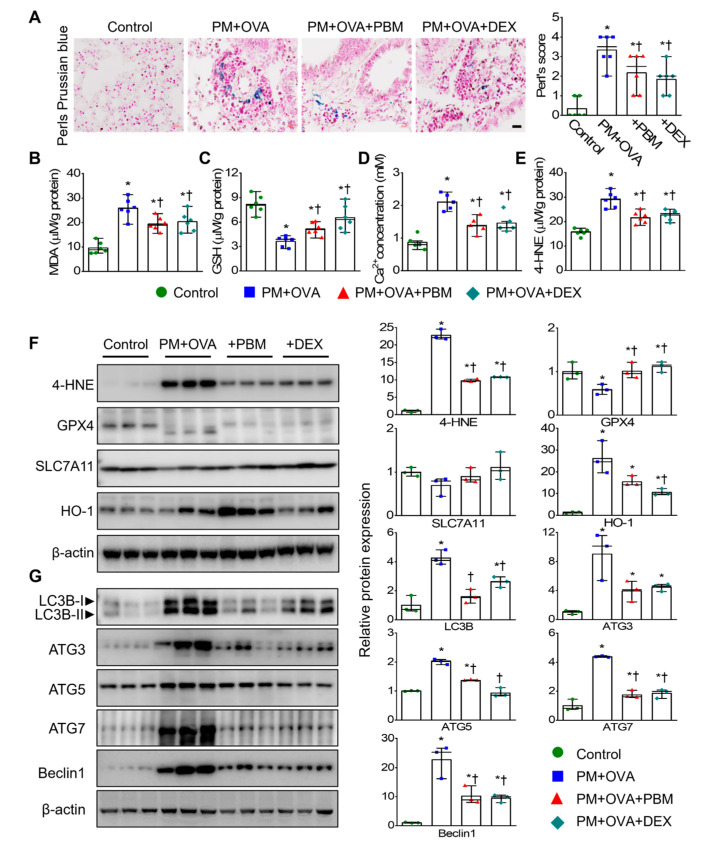
Inhibitory effects of PBM on the ferroptosis and autophagic signals in a PM_2.5_-exposed asthma exacerbation model. (**A**) Deposition of iron in lung tissue using Perls Prussian blue staining in lung tissues (*n* = 6). Scale bar represents 20 µm. (**B**) Malondialdehyde (MDA) concentration in lung tissue (*n* = 6). (**C**) Glutathione (GSH) concentration in lung tissue (*n* = 6). (**D**) Ca^2+^ levels in lung tissue (*n* = 5). (**E**) 4-Hydroxynonenal (4-HNE) levels in lung tissue (*n* = 6). (**F**) Ferroptosis markers in lung tissue (*n* = 3). (**G**) Autophagy markers in lung tissues. Bar graphs represent the quantification protein expression (*n* = 3). Data are shown as the mean ± SEM. * p < 0.05 compared to control. ^†^ *p* < 0.05 compared to PM + OVA.

The iron-dependent accumulation of lipid-ROS, lipid peroxidation, and depletion of GSH are pivotal events in ferroptosis [36]. Additionally, iron and Ca^2+^ interact through ROS signaling [37]. Therefore, we evaluated the levels of malondialdehyde (MDA), GSH, and Ca^2+^ in lung tissues. Iron accumulation and MDA levels showed a significant increase in the PM + OVA group compared to the control group. However, the treatment of PBM and DEX significantly mitigated these elevations (Figure 6A,B). Furthermore, the decreased GSH levels in the PM + OVA group were significantly restored by treatment with PBM and DEX (Figure 6C). The increased calcium levels in the PM + OVA group were significantly reduced by treatment with PBM and DEX (Figure 6D). The levels of 4-hydroxynonenal (4-HNE), a marker of ferroptosis protein, also increased in the PM + OVA group, but were notably reduced by treatment with PBM and DEX, with both treatments showing similar effects (Figure 6E). To confirm this observation, we further analyzed the expression of ferroptosis markers, including 4-HNE, GPX4, SLC7A11, and HO-1 in lung tissue. The expression levels 4-HNE and HO-1 were increased in the PM + OVA mice, while GPX4 and SLC7A11 expression was decreased; however, these alterations in protein expression were restored by treatment with PBM and DEX (Figure 6F).

In asthma, both the initiation and progression of ferroptosis involve autophagy [38]. The assessment of autophagy marker protein expression (LC3B, ATG3, ATG5, ATG7, and Beclin-1) in lung tissue showed increased levels of LC3B, ATG3, ATG5, ATG7, and Beclin-1 in PM + OVA mice. However, in mice treated with PBM, these alterations were reversed, which were similar to those treated with DEX (Figure 6G).

## 4. Discussion

This study demonstrates the promising potential of PBM therapy using a 610 nm wavelength LED as a novel and effective approach for managing allergic asthma exacerbated by PM_2.5_ exposure. By evaluating the key indicators of allergic asthma, the findings of this study revealed that PM_2.5_ exposure heightened asthma characteristics, which were significantly alleviated by PBM treatment, suggesting that PBM therapy may serve as a valuable adjunct therapy for asthma, particularly in cases worsened by environmental pollutants like PM_2.5_.

**Figure 7:**
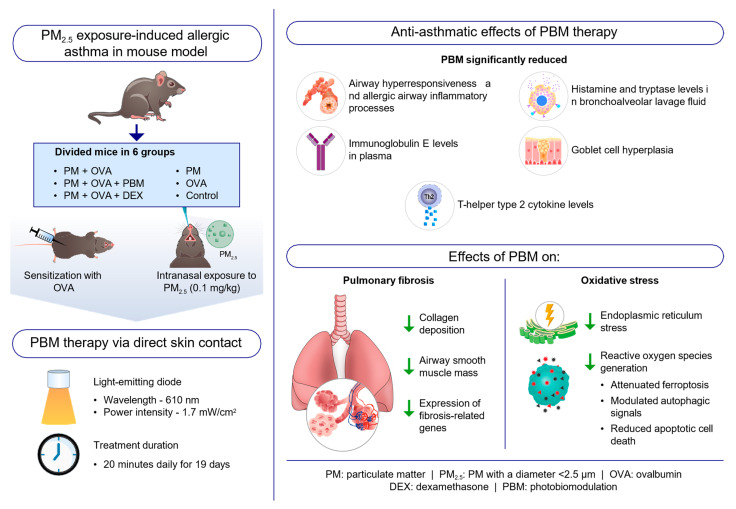
Schematic representation of the anti-asthmatic effects of photobiomodulation (PBM) therapy on PM_2.5_ exposure-induced allergic asthma in a mouse model. PBM therapy reduces AHR, inflammation, Th2 cytokines, goblet cell hyperplasia, and subepithelial fibrosis in a PM_2.5_-exacerbated allergic asthma mouse model. PBM therapy also decreases oxidative and ER stress, apoptosis, and ferroptosis, while modulating autophagy in asthmatic mice exposed to PM_2.5_. These findings suggest PBM’s potential as an adjunct to asthma treatment in patients exposed to environmental pollutants. Abbreviations: DEX, dexamethasone; OVA, ovalbumin; PBM, photobiomodulation; PM, particulate matter, PM_2.5_, PM with a diameter < 2.5 μm.

One of the key findings of this study was the significant reduction in AHR following PBM treatment. AHR is a critical characteristic of asthma, leading to bronchoconstriction and airflow limitation [39]. Dysfunctional airway smooth muscle contributes to AHR, increasing sensitivity to bronchoconstrictor stimuli [40]. Moreover, a disrupted airway epithelium drives inflammation in asthma, promoting AHR through the secretion of alarmin cytokines such as thymic stromal lymphopoietin (TSLP), IL-25, and IL-33 [41,42]. These cytokines increase the expression of Th2 cytokines, which enhance airway eosinophilia and trigger mast cells to release bronchoconstrictive mediators such as histamine, prostaglandin D2, and cysteinyl leukotrienes [42]. In our study, the significant reduction in AHR after PBM treatment indicates the potential of this therapy to address a critical characteristic of asthma that contributes to respiratory symptoms and airflow limitation. By attenuating AHR, PBM demonstrates promise in improving lung function and enhancing symptom management in patients with asthma exposed to PM_2.5_. Moreover, the decreases in eosinophilic airway inflammation, levels of Th2 cytokines, and secretion of histamine and tryptase in the BALF of PM_2.5_-exposed mice highlight the potent anti-inflammatory properties of PBM. These findings suggest that PBM can modulate immune responses and suppress inflammatory mediators, indicating its potential to alleviate airway inflammation associated with asthma exacerbations triggered by environmental factors (Figure 7).

Excessive ER stress can lead to cell death, including apoptosis, autophagy, paraptosis, and ferroptosis [43]. Extensive evidence suggests that ER stress and ROS production interact and disrupt each other, with the PERK/eIF2α signaling pathway regulating ROS production during ferroptosis [43,44]. Recent studies have highlighted the potential role of ferroptosis in asthma, a regulated cell death characterized by iron-dependent lipid peroxide accumulation and GSH depletion that leads to oxidative cell death [23,45]. In asthma, the autophagy of ferritin leads to elevated iron levels, triggered by the accumulation of ROS, contributing to airway inflammation and a hyper-oxidative state, ultimately leading to the ferroptosis of airway epithelial cells [36,46]. Oxidative stress, specifically lipid peroxidation, has been associated with asthma pathogenesis, sharing key characteristics with ferroptosis [47]. MDA levels have been shown to be elevated in the plasma [48,49] and breath condensate of individuals with asthma [50]. Furthermore, suppression of ferroptosis contributes to decreased airway inflammation [51,52] with elevated lipid peroxidation and ROS levels observed in asthma, suggesting the potential activation of ferroptosis during the progression of asthma [53].

Ferroptosis has been recognized as a type of autophagic cell death, with autophagy playing a pivotal role in triggering ferroptosis by regulating cellular iron balance and ROS generation [46,54]. In asthma, upregulated autophagy contributes to the development of Th2 immune responses and eosinophilic inflammation, while downregulated autophagy may be significant in neutrophilic asthma [55]. Consistent with previous findings, elevated levels of oxidative stress-mediated ER stress, ferroptosis, autophagy, and apoptosis-related markers were notably observed in the PM + OVA group. However, following PBM treatment, significant alterations in these processes were observed, suggesting a potential therapeutic effect of PBM in modulating these cellular mechanisms. Additionally, our investigation into the association between ferroptosis and autophagy revealed that PBM treatment reversed the alterations in expression of the autophagy markers observed in the PM + OVA group, suggesting a potential role of PBM in regulating autophagic cell death associated with ferroptosis. A schematic diagram of the anti-asthmatic effects of PBM therapy on PM_2.5_ exposure-induced allergic asthma in a mouse model is displayed in Figure 7.

While our study showed promising results, it is important to acknowledge several limitations that warrant further consideration. Our study focused on the short-term effects of PBM therapy, underscoring the need for extensive research to evaluate its long-term efficacy and safety profile in managing allergic asthma. Additionally, the precise molecular mechanisms through which PBM exerts its anti-asthmatic effects were not conclusively elucidated in our study, indicating a critical need for in-depth investigation in this area. Our study did not explore potential variations in treatment response stemming from diverse doses or durations of PBM therapy. Selecting suboptimal parameters of PBM could lead to decreased effectiveness or potentially negative treatment outcomes [56]. To better understand the precise therapeutic pathways targeting specific conditions, quantifying and standardizing light specifications, including wavelength, duration of exposure, power intensity, pulse pattern, and application time, is crucial. These limitations highlight the need for the validation of efficacy through rigorous scientific clinical trials [9]. Lastly, the generalizability of our findings to diverse patient populations or asthma phenotypes needs to be established through additional research.

## 5. Conclusions

In conclusion, PBM therapy using a 610 nm wavelength LED demonstrated significant efficacy in alleviating asthma symptoms in a mouse model of allergic airway inflammation exacerbated by PM_2.5_ exposure. The therapy effectively reduced AHR, inflammation, mucus hyperplasia, subepithelial fibrosis, and ROS-mediated ER stress as well as apoptosis, ferroptosis, and autophagy. These findings suggest that PBM therapy holds promise as an adjunct treatment for managing asthma exacerbations and shed light on the possible mechanisms underlying asthma exacerbation due to environmental pollutants. Future research should focus on validating these results in clinical trials, exploring the precise mechanisms and long-term effects, and optimizing PBM treatment parameters to facilitate its integration into clinical practice for the benefit of patients with asthma.

## Data Availability

Data are contained within the article and Supplementary Materials.

## Supplementary Materials

The following supporting information can be downloaded at: https://www.mdpi.com/article/10.3390/antiox13081003/s1, Figure S1: The raw images of the Western blot in Figure 4; Figure S2. The raw images of the Western blot in Figure 5 and Figure 6; Table S1: Primer sequences used for qRT-PCR analysis in this study.
