# Supplementary material for: Photobiomodulation Mitigates PM2.5-Exacerbated Pathologies in a Mouse Model of Allergic Asthma
Source: Antioxidants (Basel). 2024 Aug 19;13(8):1003. doi: 10.3390/antiox13081003 (PMC11351905; doi:10.3390/antiox13081003)
Supplement: Supplementary file 1 [file antioxidants-13-01003-s001.zip › antioxidants-3151173-supplementary.pdf]

Supplementary Materials

Photobiomodulation Mitigates $PM_{2.5}$-Exacerbated Pathologies in a Mouse Model of Allergic Asthma

Jisu Park [1], Bo-Young Kim [1], Eun Jung Park [1], Yong-Il Shin [1,2,*] and Ji Hyeon Ryu [1,*]

Table S1. Primer sequences used for qRT-PCR analysis in this study

Gene Name	GenBank Acc. No.	Primer Sequences (5′–3′)
Col1a1	NM_007742.4	Forward: AGACCTGTGTGTTCCCTACT<br>Reverse: GAATCCATCGGTCATGCTCTC
Col3a1	NM_009930.2	Forward: GTGACTCAGGATCTGTCCTTTG<br>Reverse: GTAGAAGGCTGTGGGCATATT
Tgfb1	NM_011577.2	Forward: GTACCTGAACCCGTGTTGCT<br>Reverse: GTATCGCCAGGAATTGTTGC
Acta2	NM_007392.3	Forward: CATGGCATCATCACCAACTG<br>Reverse: GCTGGGACATTGAAAGTCTC
Gapdh	NM_001289726.2	Forward: GTGGCAAAGTGGAGATTGTTG<br>Reverse: TTGACTGTGCCGTTGAATTTG

Col1a, type I collagen α1 chain; *Col3a*, collagen type III α1 chain; *Tgfb1*, transforming growth factor β1; *Acta2*, α-smooth muscle actin; *Gapdh*, glyceraldehyde 3-phosphate dehydrogenase

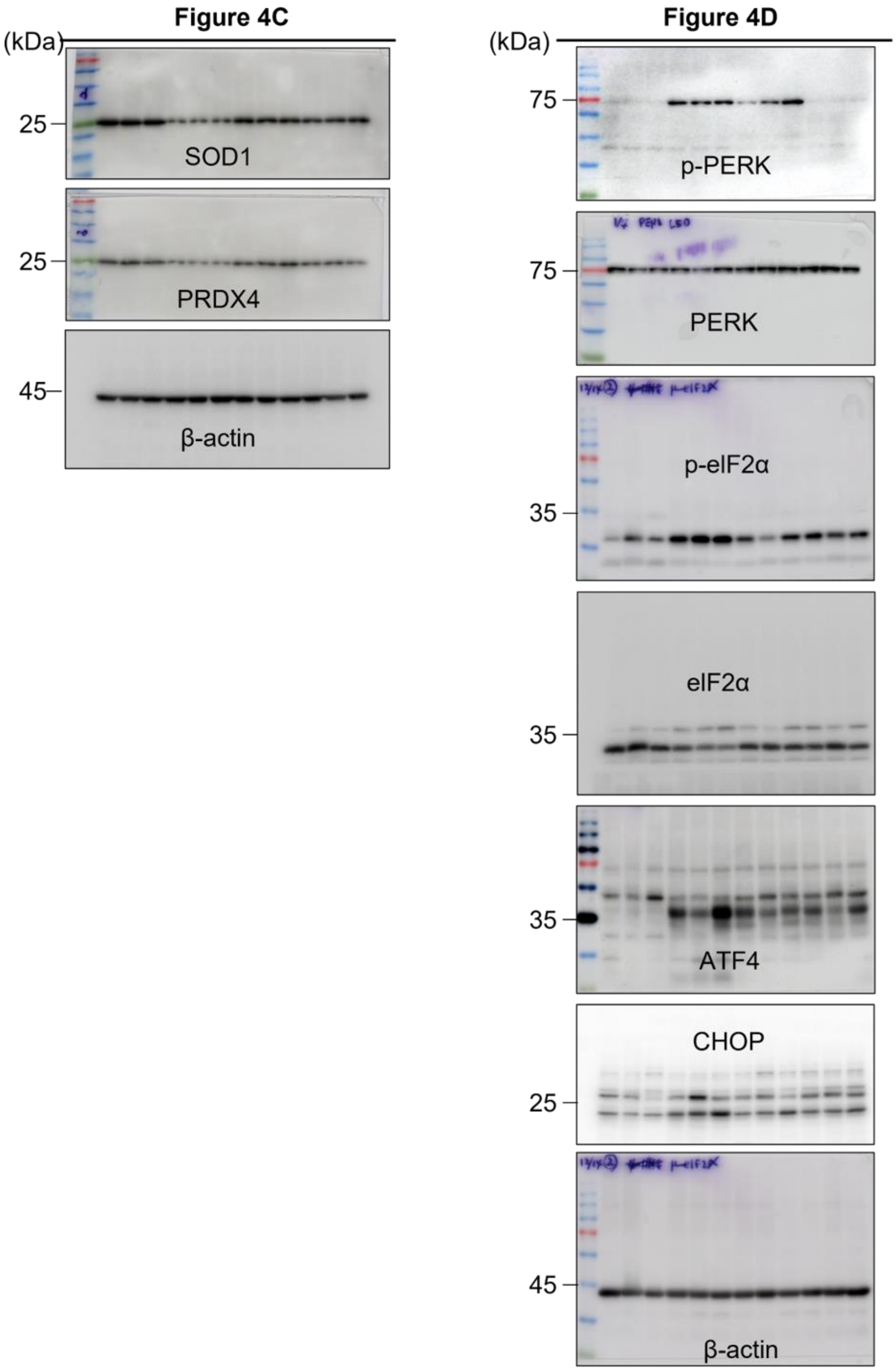

Figure S1. The raw images of the Western blot in Figure 4.

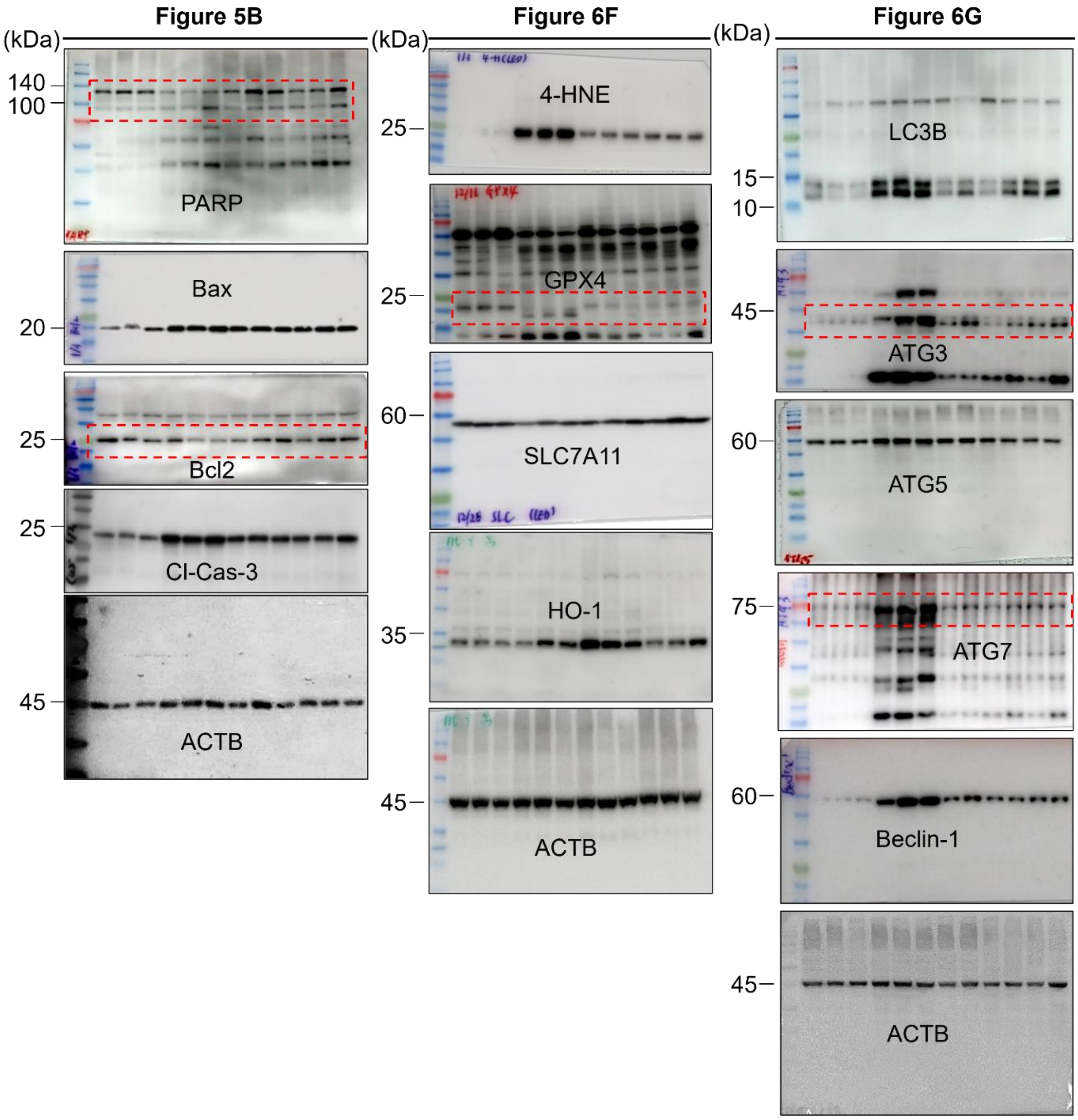

Figure S2. The raw images of the Western blot in Figure 5 and 6.
